# 异基因造血干细胞移植治疗肝炎相关性再生障碍性贫血28例疗效及安全性

**DOI:** 10.3760/cma.j.issn.0253-2727.2023.08.003

**Published:** 2023-08

**Authors:** 彦 王, 佳 李, 爱明 庞, 栋林 杨, 欣 陈, 荣莉 张, 嘉璘 魏, 巧玲 马, 卫华 翟, 祎 何, 尔烈 姜, 明哲 韩, 四洲 冯

**Affiliations:** 1 中国医学科学院血液病医院（中国医学科学院血液学研究所），北京协和医学院，实验血液学国家重点实验室，国家血液系统疾病临床医学研究中心，细胞生态海河实验室，天津 300020 State Key Laboratory of Experimental Hematology, National Clinical Research Center for Blood Diseases, Haihe Laboratory of Cell Ecosystem, Institute of Hematology & Blood Diseases Hospital, Chinese Academy of Medical Sciences & Peking Union Medical College, Tianjin 300020, China; 2 天津医学健康研究院，天津 301600 Tianjin Institutes of Health Science, Tianjin 301600, China; 3 烟台毓璜顶医院，烟台 264000 Yantai Yuhuangding Hospital, Yantai 264000, China

**Keywords:** 肝炎相关性再生障碍性贫血, 同胞全相合造血干细胞移植, 单倍体造血干细胞移植, Hepatitis related aplastic anemia, HLA-sibling allogeneic hematopoietic stem cell transplantation, Haploidentical hematopoietic stem-cell transplantation

## Abstract

**目的:**

研究异基因造血干细胞移植（allo-HSCT）治疗肝炎相关性再生障碍性贫血（HRAA）的疗效及安全性。

**方法:**

对2012年1月至2022年6月在中国医学科学院血液病医院干细胞移植中心接受allo-HSCT的HRAA患者进行回顾性分析。随访时间至2022年10月30日。

**结果:**

①共纳入接受allo-HSCT的HRAA患者28例，男18例（64.3％），女10例（35.7％），中位年龄25.5（9～44）岁。重型再生障碍性贫血（SAA）17例，极重型再生障碍性贫血（VSAA）10例，输血依赖型非重型再生障碍性贫血（TD-NSAA）1例。28例患者中单倍体造血干细胞移植（haplo-HSCT）15例，同胞全相合造血干细胞移植（MSD-HSCT）13例。②移植后100 d Ⅱ～Ⅳ度急性GVHD累积发生率为25.0％（95％*CI* 12.8％～45.4％），2年慢性GVHD累积发生率为4.2％（95％*CI* 0.6％～25.4％）。③移植后2年总生存（OS）率为81.4％（95％*CI* 10.5％～20.6％），无失败生存（FFS）率为81.4％（95％*CI* 10.5％～20.6％），移植相关死亡率（TRM）为14.6％（95％*CI* 5.7％～34.3％），所有患者移植后均未发生显著肝损伤。④haplo-HSCT组移植后巨细胞病毒（CMV）血症发生率高于MSD-HSCT组［60.0％（95％*CI* 35.2％～84.8％）对7.7％（95％*CI* 0～22.2％），*P*＝0.004］，两组EB病毒血症发生率、2年OS率、2年FFS率、2年TRM、移植后100 d Ⅱ～Ⅳ度急性GVHD累积发生率及2年慢性GVHD累积发生率差异均无统计学意义。

**结论:**

allo-HSCT治疗HRAA安全有效。在无法获得HLA全相合同胞供者时，haplo-HSCT可作为替代选择。

## Abstract (en)

**Objective:**

To evaluate the efficacy and safety of HLA-haploidentical hematopoietic stem cell transplantation（allo-HSCT）for hepatitis-related aplastic anemia（HRAA）patients.

**Methods:**

Retrospective analysis was performed on hepatitis-associated aplastic anemia patients who received haplo-HSCT at our center between January 2012 and June 2022. October 30, 2022 was the final date of follow-up.

**Results:**

This study included 28 HRAA patients receiving allo-HSCT, including 18 males（64.3％）and 10 females（35.7％）, with a median age of 25.5（9–44）years. About 17 cases of severe aplastic anemia（SAA）, 10 cases of very severe aplastic anemia（VSAA）, and 1 case of transfusion-dependent aplastic anemia（TD-NSAA）were identified. Among 28 patients, 15 patients received haplo-HSCT, and 13 received MSD-HSCT. The 2-year overall survival（OS）rate, the 2-year failure-free survival（FFS）rate, the 2-year transplant-related mortality（TRM）rate, the 100-day grade Ⅱ–Ⅳ acute graft-versus-host disease（aGVHD）cumulative incidence rate, and the 2-year chronic graft-versus-host disease（cGVHD）cumulative incidence rate were 81.4％, 81.4％（95％*CI* 10.5％–20.6％）, 14.6％（95％*CI* 5.7％–34.3％）, 25.0％（95％*CI* 12.8％–45.4％）, and 4.2％（95％*CI* 0.6％–25.4％）, respectively. After transplantation, all patients had no significant liver function damage. Compared with the MSD-HSCT group, only the incidence of cytomegaloviremia was significantly higher in the haplo-HSCT group [60.0％（95％*CI* 35.2％–84.8％）*vs* 7.7％（95％*CI* 0–22.2％）, *P*＝0.004]. No statistically significant difference in the Epstein-Barr virus was found in the 2-year OS, 2-year FFS, 2-year TRM, and 100-day grade Ⅱ–Ⅳ aGVHD cumulative incidence rates and 2-year cGVHD cumulative incidence rate.

**Conclusion:**

Allo-HSCT is safe and effective for HRAA, and haplo-HSCT can be used as a safe and effective alternative for newly diagnosed HRAA patients who cannot obtain HLA-matched sibling donors.

肝炎相关性再生障碍性贫血（HRAA）是获得性再生障碍性贫血（AA）的一种罕见亚型，占初诊AA的1％～5％，亚洲发病率高于欧美[1]，儿童较成人多见[2]，男性多于女性。其特征是通常在血清转氨酶升高6个月之内出现全血细胞减少伴骨髓增生不良或造血功能衰竭[3]。HRAA的一线治疗方案包括免疫抑制治疗（IST）和异基因造血干细胞移植（allo-HSCT）。对于重型及输血依赖非重型的HRAA患者，首选IST或allo-HSCT尚无定论。本研究对在本中心接受allo-HSCT的28例HRAA患者进行回顾性分析，旨在探究allo-HSCT治疗HRAA的疗效及安全性并对同胞全相合造血干细胞移植（MSD-HSCT）与单倍体造血干细胞移植（haplo-HSCT）两种移植模式在HRAA患者中的疗效进行比较。

## 病例与方法

一、病例

本研究纳入2012年1月至2022年6月在中国医学科学院血液病医院造血干细胞移植中心接受allo-HSCT的28例HRAA患者，对其临床资料进行回顾性分析。所有患者均知情同意。

二、移植预处理及移植物抗宿主病（GVHD）预防

所有患者均接受抗胸腺细胞球蛋白（ATG）+环磷酰胺（Cy）+氟达拉滨（Flu）±白消安（Bu）的预处理方案；具体剂量：Flu 30 mg·m^−2^·d^−1^×5 d（−5 d～−1 d），Cy 37.5 mg·m^−2^·d^−1^×3 d或50 mg·m^−2^·d^−1^× 3 d，抗人T细胞猪免疫球蛋白（p-ATG）20～25 mg·kg^−1^·d^−1^×5 d或兔抗人胸腺细胞免疫球蛋白（r-ATG）2.5 mg·kg^−1^·d^−1^×5 d，Bu 3.2 mg·kg^−1^·d^−1^×2 d。所有患者均接受环孢素A（CsA）或他克莫司（FK506）+甲氨蝶呤（MTX）±霉酚酸酯（MMF）方案预防GVHD。

三、支持治疗

所有患者在移植期间均住层流病房，直到移植后中性粒细胞数恢复。移植前常规口服复方磺胺甲恶唑和阿苯达唑，部分患者移植前应用缬更昔洛韦或膦甲酸钠预防巨细胞病毒（CMV）。对于移植前无侵袭性真菌病（IFD）病史的患者，给予氟康唑一级预防，至移植后3个月停药。移植前患有IFD的患者给予伊曲康唑、伏立康唑、米卡芬净或卡泊芬净二级预防。

血小板计数低于20×10^9^/L或有活动性出血的患者，予以血小板输注；血红蛋白低于70 g/L的患者，予以红细胞输注。从移植后第6天开始应用粒细胞集落刺激因子（G-CSF），直到中性粒细胞计数（ANC）恢复正常。

所有患者在移植住院期间每周进行3次CMV和EB病毒检测，出院后每周检测1次，直到移植后6个月。

四、定义及标准

本组病例再生障碍性贫血（AA）的的诊断均符合《再生障碍性贫血诊断与治疗中国专家共识（2017年版）》[4]；HRAA定义为患者血细胞减少与骨髓造血功能衰竭与急性肝功损害（血清转氨酶水平升高达到正常5倍以上）同时发生或在肝功损害后6个月内发生，血清嗜肝性病毒检测均为阴性[5]。急性GVHD（aGVHD）、慢性GVHD（cGVHD）的诊断和分级标准参照2020年EBMT共识[6]；IFD的诊断参照EORTC/MSG共识[7]。ANC≥0.5×10^9^/L连续3 d为粒细胞植入，血小板计数≥20×10^9^/L连续7 d且脱离血小板输注为血小板植入。本研究将菌血症和重症肺炎定义为严重感染。

五、随访

通过查阅门诊/住院病历及电话随访的方式获取患者移植后生存情况。随访截止日期为2022年10月30日。移植后中位随访时间为1 091（20～3 024）d。观察指标包括总生存（OS）、无失败生存（FFS）、移植相关死亡率（TRM）。OS定义为从移植之日到因任何原因死亡之日或末次随访的时间。FFS定义为从移植之日到失败事件（原发或继发植入失败、复发、死亡等）发生的时间。

六、统计学处理

应用SPSS 26及R 4.2.2进行数据分析。分类变量组间比较采用卡方检验或Fisher精确检验，连续变量组间比较采用*t*检验（不符合正态分布）或Mann-Whitney *U*检验（不符合正态分布）。使用竞争风险模型估计事件的累计发生率，使用Gray's检验比较组间差异。意外死亡及复发死亡为TRM的竞争事件；死亡为GVHD的竞争事件。用Kaplan-Meier生存分析评估累计生存率，用Log-rank检验比较组间差异。*P*<0.05认为具有统计学意义。

## 结果

一、患者基线特征

本研究共回顾性纳入28例接受allo-HSCT的HRAA患者，确诊时血清嗜肝性病毒（甲肝、乙肝、丙肝、戊肝）抗体和DNA/RNA检测均为阴性。haplo-HSCT组15例，MSD-HSCT组13例。28例患者中男18例（64.3％）、女10例（35.7％），中位发病年龄为25.5（9～44）岁。重型再生障碍性贫血（SAA）17例，极重型再生障碍性贫血（VSAA）10例，输血依赖性非重型再生障碍性贫血（TD-NSAA）1例。移植前血常规（中位数）：WBC 1.22（0.11～4.27）×10^9^/L、ANC 0.27（0.01～1.37）×10^9^/L、HGB 80.5（45～143）g/L、PLT 14.5（1～64）×10^9^/L、网织红细胞8.8（1.8～112.9）×10^9^/L。全部28例患者起病时均有肝功能异常，中位丙氨酸氨基转移酶（ALT）1 365（254～2 800）U/L，中位天门冬氨酸氨基转移酶（AST）895（69～1 800）U/L；从发现肝功能异常到检出血细胞减少的时间为2（0～7）个月；所有患者移植前肝功能均恢复正常。1例（3.8％）患者合并PNH克隆（粒细胞PNH克隆1％，单核细胞PNH克隆1.5％）。

共有6例（21.4％）患者移植前存在感染，MDS-HSCT组、haplo-HSCT组各3例。MDS-HSCT组3例（23.1％）患者均为肺部真菌感染（拟诊），其中1例患者合并铜绿假单胞菌血症；haplo-HSCT组3例（20％）患者有肺部感染，其中1例患者经支气管肺泡灌洗液检查确诊肺毛霉菌感染，2例患者为肺真菌感染（拟诊）。均给予相应抗细菌抗真菌药物治疗。

HRAA诊断与allo-HSCT的间隔时间为2.7（1.1～17.0）个月。MSD-HSCT组2例（7.1％）患者移植前ATG +CsA治疗无效，其余患者移植前均以CsA维持治疗。两组患者临床特征比较差异无统计学意义（详见表1）。

**表1:** 28例接受异基因造血干细胞移植治疗肝炎相关性再生障碍性贫血患者的基线资料

指标	总体（28例）	haplo-HSCT组（15例）	MSD-HSCT组（13例）	*P*值
性别［例（%）］				
男	18（64.3）	10（66.7）	8（61.5）	1.000
女	10（35.7）	5（33.3）	5（38.5）	1.000
发病年龄［岁，*M*（范围）］	25.5（9~44）	25（9~38）	26（12~44）	0.821
诊断［例（%）］				0.836
SAA	17（60.7）	9（60.0）	8（61.5）	
VSAA	10（35.7）	6（40.0）	4（30.8）	
TD-NSAA	1（3.6）	0	1（7.7）	
移植前IST［例（%）］				0.206
是	2（7.1）	0	2（15.4）	
否	26（92.9）	15（100.0）	11（84.6）	
初诊肝功能［U/L，*M*（范围）］				
ALT	1 365（254~2 800）	1 312（254~2 500）	1 400（261~2 800）	0.400
AST	895（69~1 800）	932（69~1 800）	484（124~1 144）	0.392
肝功异常到发生血细胞减少［月，*M*（范围）］	2.0（0~7.0）	2.0（0~5.0）	2.0（0~7.0）	0.856
确诊到移植［月，*M*（范围）］	2.7（1.1~17.0）	2.5（1.1~10.7）	2.8（1.5~17.0）	0.525
移植前感染［例（%）］	6（21.4）	3（20.0）	3（23.1）	0.843
血常规［*M*（范围）］				
WBC（×10^9^/L）	1.22（0.11~4.27）	1.19（0.11~4.27）	1.25（0.14~1.96）	0.464
ANC（×10^9^/L）	0.27（0.01~1.37）	0.27（0.01~0.92）	0.29（0.01~1.37）	0.348
HGB（g/L）	80.5（45~143）	82（66~134）	76（45~143）	0.284
PLT（×10^9^/L）	14.5（1~64）	16（1~43）	14（1~64）	0.576
网织红细胞（×10^9^/L）	8.8（1.8~112.9）	6.6（1.8~112.9）	10.5（3.5~65.0）	0.871

**注** haplo-HSCT：单倍体造血干细胞移植；MSD-HSCT：同胞全相合造血干细胞移植；SAA：重型再生障碍性贫血：VSAA：极重型再生障碍性贫血；TD-NSAA：输血依赖性非重型再生障碍性贫血；IST：免疫抑制治疗；ALT：丙氨酸氨基转移酶；AST：天门冬氨酸氨基转移酶；ANC：中性粒细胞计数

二、移植过程

移植物包括外周血干细胞、骨髓、外周血干细胞+骨髓；预处理方案的选择：共有6例（21.4％）患者应用Bu+Cy+Flu+ATG预处理方案、22例（78.6％）应用Cy+Flu+ATG预处理方案；GVHD的预防用药有FK506/CsA+MTX±MMF。haplo-HSCT组单个核细胞（MNC）、CD34^+^细胞的中位输注量分别为10.17（7.79～16.94）×10^8^/kg、3.13（1.59～4.62）×10^6^/kg，MSD-HSCT组MNC、CD34^+^细胞的中位输注量分别为10（6.65～30.52）×10^8^/kg、2.42（1.88～6.10）×10^6^/kg。28例HRAA患者移植相关资料详见表2。

**表2:** 28例肝炎相关性再生障碍性贫血患者异基因造血干细胞移植相关资料

指标	总体（28例）	haplo-HSCT（15例）	MSD-HSCT（13例）	*P*值
移植物来源［例（%）］				1.000
PBSC	23（82.1）	12（80.0）	11（84.6）	
PBSC+BM	3（3.0）	2（13.3）	1（7.7）	
BM	2（7.1）	1（6.7）	1（7.7）	
供患者ABO血型匹配［例（%）］				0.198
匹配	7（25.0）	13（86.7）	8（61.5）	
不匹配	21（75.0）	2（13.3）	5（38.5）	
供患者性别组合［例（%）］				0.439
女供女	5（17.9）	1（6.7）	4（30.8）	
女供男	6（21.4）	3（20）	3（23.1）	
男供男	11（39.3）	7（46.7）	4（30.8）	
男供女	6（21.4）	4（26.6）	2（15.3）	
MNC［×10^8^/kg，*M*（范围）］	10.08（6.65~30.52）	10.17（7.79~16.94）	10.00（6.65~30.52）	0.988
CD34^+^细胞［×10^6^/kg，*M*（范围）］	2.73（1.59~6.10）	3.13（1.59~4.62）	2.42（1.88~6.10）	0.328
预处理方案［例（%）］				0.655
Bu+Cy+Flu+ATG	6（21.4）	4（26.6）	2（15.3）	
Cy+Flu+ATG	22（78.6）	11（73.4）	11（84.7）	
GVHD预防［例（%）］				<0.001
CsA+MTX+MMF	10（35.7）	10（66.7）	0	
FK506+MTX+MMF	2（7.2）	2（13.3）	0	
CsA+MTX	13（46.4）	2（13.3）	11（84.7）	
FK506+MTX	3（10.7）	1（6.7）	2（15.3）	

**注** haplo-HSCT：单倍体造血干细胞移植；MSD-HSCT：同胞全相合造血干细胞移植；PBSC：外周血干细胞；BM：骨髓；MNC：单个核细胞；Bu：白消安；Cy：环磷酰胺；Flu：氟达拉滨；ATG：抗胸腺细胞球蛋白；CsA：环孢素A；MTX：甲氨蝶呤；MMF：霉酚酸酯；FK506：他克莫司；GVHD：移植物抗宿主病

三、造血重建和GVHD

1例（6.7％）haplo-HSCT组患者血小板原发性植入失败并死于重症感染，其余27例患者移植后均获得中性粒细胞和血小板植入并存活超过28 d。haplo-HSCT组与MSD-HSCT组中性粒细胞植入中位时间分别为12（11～20）d、12（11～15）d，血小板植入中位时间分别为16（9～45）d、13（8～32）d。

移植后100 d Ⅱ～Ⅳ度aGVHD累积发生率为25.0％（95％*CI* 12.8％～45.4％），2年cGVHD累积发生率为4.2％（95％*CI* 0.6％～25.4％）。haplo-HSCT组中9例（60.0％）患者发生aGVHD；MSD-HSCT组中2例（15.4％）患者发生aGVHD，1例（7.7％）患者发生cGVHD。haplo-HSCT组、MSD-HSCT组移植后100 d Ⅱ～Ⅳ度aGVHD的发生率分别为33.3％（95％*CI* 15.4％～62.5％）、15.4％（95％*CI* 4.1％～48.8％）（*P*＝0.247）。haplo-HSCT组、MSD-HSCT组移植后cGVHD的发生率分别为0、8.3％（95％*CI* 1.2％～46.1％）（*P*＝0.283）。GVHD涉及的器官有皮肤、肠道、肺、口腔、膀胱。

四、治疗相关毒性和感染

大多数患者移植过程中耐受性良好。2组中各有1例患者在移植后40 d左右发生Ⅱ度aGVHD（肝脏1级），未发生Ⅲ/Ⅳ级肝毒性、药物性肝损伤及肝窦阻塞综合征（SOS）等严重肝脏事件。

移植100 d内，haplo-HSCT组、MSD-HSCT组CMV血症发生率分别为60.0％（95％*CI* 35.2％～84.8％）、7.7％（95％*CI* 0～22.2％）（*P*＝0.004），EB病毒血症的发生率分别为20.0％（95％*CI* 0～40.2％）、0（*P*＝0.080）。haplo-HSCT组4例（26.7％）、MSD-HSCT组1例（7.7％）患者发生菌血症，致病菌包括大肠埃希菌（CRE）、肺炎克雷伯菌、铜绿假单胞菌；haplo-HSCT组1例（6.7％）患者移植后出现肺真菌感染（拟诊）。

五、随访及生存情况

至2022年10月30日，28例患者中有23例（82.1％）存活。移植后2年OS率为81.4％（95％*CI* 10.5％～20.6％），FFS率为81.4％（95％*CI* 10.5％～20.6％），TRM为14.6％（95％*CI* 5.7％～34.3％）。MSD-HSCT组2例（15.4％）死亡，其中1例死于aGVHD，1例死于意外事故；haplo-HSCT组3例（20％）死亡，死亡原因分别为aGVHD、重症感染、多脏器功能衰竭。haplo-HSCT组与MSD-HSCT组中位随访时间分别为1 429（23～3 212）d、786（58～3 078）d，TRM分别为20.6％（95％*CI* 7.1％～51.2％）、7.7％（95％*CI* 1.1％～43.4％）（*P*＝0.313）。移植后2年OS率分别为79.4％（95％*CI* 61.2％～100％）、83.1％（95％*CI* 64.1％～100％）（图1A，*P*＝0.665），FFS率分别为79.4％（95％*CI* 61.2％～100％）、83.1％（95％*CI* 64.1％～100％）（图1B，*P*＝0.617）。

**图1:**
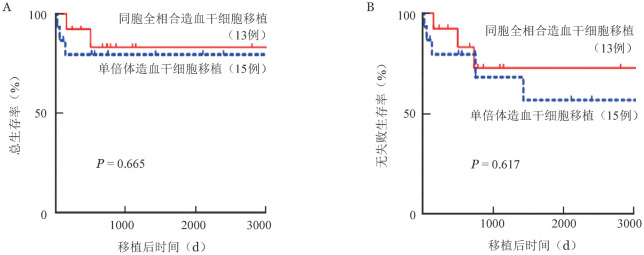
单倍体造血干细胞移植组、同胞全相合造血干细胞移植组移植后总生存曲线（A）与无失败生存曲线（B）

## 讨论

HRAA是AA中一种罕见的特殊亚型，多见于青少年男性[8]。HRAA起病急骤，病死率高达78％～88％，严重感染及内脏出血是主要的致死原因[9]–[10]。患者多在急性肝炎发作3～6个月内出现骨髓造血功能衰竭、全血细胞减少[3],[8]。肝炎症状可表现为乏力、黄疸、血清转氨酶升高等，大部分患者在发现血细胞减少前肝功能可部分或完全恢复正常[9]。尽管多种嗜肝病毒均可导致骨髓造血功能减低甚至造血功能衰竭，但大多数HRAA患者血清相关嗜肝病毒检测均为阴性，因此也被称为“血清学阴性的肝炎相关性再生障碍性贫血”[3]。本组28例患者中男性占65.5％，中位发病年龄为25.5（9～44）岁。肝功能异常间隔与全血细胞减少的时间为2.0（0～7）个月，所有患者均未检出嗜肝病毒。

与AA相似，HRAA的一线治疗多采用IST与allo-HSCT。对于重型及输血依赖性非重型的HRAA患者首选治疗方案尚无定论。Osugi等[11]研究发现儿童HRAA患者接受ATG和CsA治疗6个月后的总缓解率为70.4％，10年OS率为88.3％。本中心以往研究显示，与接受IST的获得性AA相比，HRAA患者感染控制所需时间明显较长［21（4～100）d对13（3～139）d，*P*＝0.048］，但3个月（34.1％对34.1％，*P*＝1.000）、6个月（56.1％对53.7％，*P*＝0.787）及12个月（73.2％对68.3％，*P*＝0.558）血液学反应率差异均无统计学意义[12]–[13]。EMBT的研究显示一线接受IST的HRAA患者10年OS率为68％，而一线接受MSD-HSCT的患者10年OS率为73％[8]。本研究中2例患者在移植前ATG+CsA治疗无效，28例HRAA患者移植后的总缓解率为82.1％（23/28），2年OS率为81.4％（95％*CI* 10.5％～20.6％），2年TRM为14.6％（95％*CI* 5.7％～34.3％），移植后100 d Ⅱ～Ⅳ度aGVHD累积发生率为25.0％（95％*CI* 12.8％～45.4％），2年cGVHD累积发生率为4.2％（95％*CI* 0.6％～25.4％），且所有患者移植后均无显著肝损伤，提示allo-HSCT治疗HRAA安全有效。

以往研究表明，allo-HSCT后CMV再激活率为40％～60％，患者的年龄及性别、供者来源、供者和患者的CMV血清状态、年龄以及应用ATG、单倍体移植、Ⅱ～Ⅳ度aGVHD等因素均可增加CMV再激活的风险[14]–[16]。本研究haplo-HSCT组CMV再激活率高于MSD-HSCT组，与上述研究结果一致。

目前国内外指南均推荐MSD-相合同胞供者的SAA患者不足30％，因此haplo-HSCT、无关供者造血干细胞移植（UD-HSCT）、脐血干细胞移植等也可作为替代选择[20]–[21]。EBMT的研究证实，与获得性AA相比，接受MSD-HSCT的HRAA患者移植后10年OS率无明显差异[8]。由于移植后免疫重时间延迟，haplo-HSCT后GVHD、重症感染的发生率较MSD-HSCT明显增高，可影响移植效果、甚至引发患者死亡；但随着预处理方案及支持治疗等改善，haplo-HSCT治疗AA的疗效也逐渐提高。Ma等[22]应用haplo-HSCT一线治疗15例HRAA患者，移植后3年OS率达100％，治疗相关毒性、GVHD发生率、OS率与非HRAA组差异无统计学意义。本研究中虽然haplo-HSCT组移植后CMV感染发生率高于MSD-HSCT组，但OS、FFS、TRM、Ⅱ～Ⅳ度aGVHD及cGVHD累积发生率差异均无统计学意义，显示haplo-HSCT与MSD-HSCT在治疗HRAA的安全性及有效性方面并无差异。

综上，本组病例结果显示，allo-HSCT是HRAA安全有效的治疗方法，对于无HLA匹配同胞供者的HRAA患者，haplo-HSCT可作为替代选择。
